# Genome-Wide Association Analysis Reveals Trait-Linked Markers for Grain Nutrient and Agronomic Traits in Diverse Set of Chickpea Germplasm

**DOI:** 10.3390/cells11152457

**Published:** 2022-08-08

**Authors:** Rajasekhar Srungarapu, Mahesh Damodhar Mahendrakar, Lal Ahamed Mohammad, Uttam Chand, Venkata Ramana Jagarlamudi, Kiran Prakash Kondamudi, Himabindu Kudapa, Srinivasan Samineni

**Affiliations:** 1 Accelerated Crop Improvement, Chickpea Breeding, International Crops Research Institute for the Semi-Arid Tropics, Patancheru 502324, India; 2 Department of Molecular Biology and Biotechnology, Acharya N.G. Ranga Agricultural University, Guntur 522034, India; 3 Department of Genetics and Plant Breeding, Acharya N.G. Ranga Agricultural University, Guntur 522034, India; 4 Department of Statistics and Computer Applications, Acharya N.G. Ranga Agricultural University, Guntur 522034, India; 5 Genomics, Pre-Breeding and Bioinformatics, International Crops Research Institute for the Semi-Arid Tropics, Patancheru 502324, India

**Keywords:** chickpea reference set, grain Fe, Zn, protein, linkage disequilibrium, population structure, GWAS, FarmCPU, BLINK

## Abstract

Chickpea is an inexpensive source of protein, minerals, and vitamins to the poor people living in arid and semi-arid regions of Southern Asia and Sub-Saharan Africa. New chickpea cultivars with enhanced levels of protein, Fe and Zn content are a medium-term strategy for supplying essential nutrients for human health and reducing malnutrition. In the current study, a chickpea reference set of 280 accessions, including landraces, breeding lines, and advanced cultivars, was evaluated for grain protein, Fe, Zn content and agronomic traits over two seasons. Using a mid-density 5k SNP array, 4603 highly informative SNPs distributed across the chickpea genome were used for GWAS analysis. Population structure analysis revealed three subpopulations (K = 3). Linkage disequilibrium (LD) was extensive, and LD decay was relatively low. A total of 20 and 46 marker-trait associations (MTAs) were identified for grain nutrient and agronomic traits, respectively, using FarmCPU and BLINK models. Of which seven SNPs for grain protein, twelve for Fe, and one for Zn content were distributed on chromosomes 1, 4, 6, and 7. The marker S4_4477846 on chr4 was found to be co-associated with grain protein over seasons. The markers S1_11613376 and S1_2772537 co-associated with grain Fe content under NSII and pooled seasons and S7_9379786 marker under NSI and pooled seasons. The markers S4_31996956 co-associated with grain Fe and days to maturity. SNP annotation of associated markers were found to be related to gene functions of metal ion binding, transporters, protein kinases, transcription factors, and many more functions involved in plant metabolism along with Fe and protein homeostasis. The identified significant MTAs has potential use in marker-assisted selection for developing nutrient-rich chickpea cultivars after validation in the breeding populations.

## 1. Introduction

Micronutrient and protein malnutrition or hidden hunger is widely spread in developing countries, especially among poor populations living in arid and semi-arid regions of Southern Asia and Sub-Saharan Africa. As a result of the growing consumption of calorie-dense foods, the globe is confronting a serious nutrient deficiency crisis that directly threatens nutritional security. Protein and micronutrients are essential components of a healthy diet for proper growth and development. Proteins are involved in various metabolic processes, biological functions, and immunological defense systems in biological cells. They act as enzymes, hormones, immunoglobulins, and structural elements [1]. Micronutrients are ingested in meagre quantities; however, if they are not consumed, they can create deficiencies, and particularly Fe and Zn deficiencies could lead to poor health, increased mortality, and learning disabilities. Fe and Zn deficiencies are more common, affecting more than 20~30% of the population globally and causing poor immunological functions, anemia in pre-school children and pregnant women [2,3,4,5]. A sustainable solution would be linking agriculture to nutrition and health, i.e., biofortification [6]. Several crop varieties fortified with different nutrients are being cultivated globally to cater the problems associated with malnutrition [7,8,9]. Recently, the government of India raised a campaign to promote bio-fortified varieties of staple crops for achieving nutritional security in the country [10].

Chickpea is the second most important pulse crop of profound economic and nutritional value. It provides an inexpensive and quality source of dietary proteins, complex carbohydrates, vitamins, and micronutrients required for human nutrition [11,12,13,14]. As chickpea is consumed in various forms in different ecologies, it serves as an essential nutritional supplement to the poor people living in the drylands of Asia and Africa. Recent studies showed the existence of a large variability for grain protein, Fe and Zn in chickpea germplasm, breeding lines, and cultivars [14,15], which opened ways to understand the genetic architecture and inheritance pattern of the traits for devising breeding strategies. A large variability for grain protein (16.3–26.2%), Fe (44.1–76.7 mg kg^−1^), and Zn (36.3–56.2 mg kg^−1^) contents with moderate-to-high heritability was observed among 280 chickpea accessions used in the current study [16]. Improved chickpea cultivars with enhanced levels of protein and Fe and Zn content contribute towards the reduction of nutrient deficiency and assure global food and nutritional security.

Grain nutrients accumulation is a complex process that is controlled by several genes and influenced by environmental factors. GWAS is an efficient approach for mapping biologically and economically important traits in diverse genetic populations at a higher resolution than bi-parental mapping. GWAS was used extensively to characterize the extent of genetic variation and uncover specific genes underlying traits and identification of marker-trait associations for agronomic, biotic, abiotic, and nutritional traits in chickpea [17,18,19,20,21,22,23] and other legumes such as lentil [24], soybean [25], and pea [26]. In chickpea, MTAs for nutrient traits under normal versus heat- and drought-stress conditions were identified [11,15,27,28,29]; however, these markers need to be validated for use in the marker-assisted selection programs to improve the selection efficiency in the breeding pipeline. In the current study, efforts were also made to compare the MTAs identified in the previous study [15] to identify more loci-controlling nutrients. Based on the aforesaid, limited informative markers linked to grain nutrient traits were available in chickpea. In this context, the current study was undertaken to delineate SNP variants regulating grain nutrient and agronomic traits via GWAS analysis using FarmCPU and BLINK models in the chickpea reference set.

## 2. Materials and Methods

### 2.1. Plant Material

The experimental material for the present investigation comprised of 280 accessions representing diverse global chickpea reference set maintained at ICRISAT Gene Bank, which includes 260 landraces, 5 advanced cultivars, 11 breeding materials, and 4 accessions with unknown biological status from 31 different geographical locations (Supplementary Table S1) was evaluated for protein, Fe and Zn content under normal season1 (NSI) and normal season2 (NSII). The experiment was conducted on vertisols in an alpha-lattice design with three replications at ICRISAT (17°30′ N; 78°16′ E; altitude 549 m), Patancheru, India. Each accession was grown in an area of 1.2 m^2^ plot with 60 × 10 cm inter- and intra-row spacing. The field experiments were conducted during the third week of November 2019 (NSI) and the second week of November 2020 (NSII). Two irrigations were provided: one at the initial stage of the planting to ensure uniform germination and another at the podding stage. For successful crop establishment in each season, standard agronomic practices were followed. Agronomic observations such as days to 50% flowering (DFF), days to maturity (DM), and 100 seed weight (100 SW) were recorded in each plot.

### 2.2. Estimation of Grain Protein, Fe and Zn

Mineral analysis (Fe and Zn content) and protein content estimation of grain samples were conducted using ICP-OES (Inductively Coupled Plasma–Optical Emission Spectrometry) and Kjeldahl methods, respectively. To avoid soil contamination, seeds from each accession and replication were harvested separately in cloth bags and threshed manually to avoid metal contamination. 10–15 g seeds were cleaned with a cloth to avoid further dust contamination and then transferred to paper bags for protein, Fe and Zn content estimation. Details of the grain sample analyses were followed as reported in earlier studies in chickpea [15,30].

### 2.3. DNA Isolation and Quantification

The genomic DNA was extracted from young leaf tissues (10 to 15 days old) of chickpea accessions at the seedling stage using the QIAGEN DNeasy 96 plant kit and quantified using a NanoDrop ND-1000 spectrophotometer (Thermo Scientific, Wilmington, NC, USA).

### 2.4. SNP Genotyping and Filtering for GWAS

The 280 accessions were genotyped using mid-density 5K SNP panel [31]. The 4995 SNP arrays were filtered for minor allele frequency (MAF > 0.05), maximum missing sites per SNP < 30%, and heterozygosity of 0.1%. A total of 4603 SNPs were selected on eight chromosomes of chickpea and used to determine the genetic diversity, population structure, LD and marker-trait associations for nutrient (grain protein, Fe and Zn), and agronomic traits (DFF, DM, and 100 SW) in this study.

### 2.5. Analysis of Population Structure and Linkage Disequilibrium

Bayesian clustering approach was implemented using STRUCTURE 2.3.4 tool to investigate the subpopulation structure based on an “admixture” model [32]. It is a model-based clustering algorithm to identify genetic clusters in the form of K (sub-population) values. The analysis was performed in multiple runs arranging successive values of K from 2 to 10 with a burn-in period set at 10,000 and 1,00,000 Markov-chain Monte Carlo (MCMC) repetitions with 3 iterations. The optimum K value was determined based on the ∆(K) method extracted using STRUCTURE HARVESTER [33,34]. An unweighted neighbor-joining (NJ) tree was constructed based on a dissimilarity matrix (DM) estimated from the 4603 SNPs by TASSEL 5.2.80 [35]. The genome-wide linkage disequilibrium (LD) was generated by plotting average *r^2^* (correlation frequency among SNPs) values as a function of genetic distance in base pairs (bp) against eight chromosomes across the chickpea genome using the TASSEL 5.2.80. LD decay plot was imputed in R (https://www.r-project.org) (accessed on 3 February 2022) and PCA by using packages “factoextra”, “Factominer”, and “tidyverse” in R version 4.1.2.

### 2.6. Association Mapping–GWAS for Grain Nutrient and Agronomic Traits

Best linear unbiased predictors (BLUPs) were estimated for all the traits from NSI, NSII, and combined analysis. Marker-trait associations (MTAs) were identified under normal and pooled seasons by using different GAPIT models. BLINK (Bayesian-information and Linkage-disequilibrium Iteratively Nested Keyway) and FarmCPU (Fixed and Random Model Circulating Probability Unification) models were used to evaluate the marker-trait associations with K (kinship) values and principal coordinate values as covariates in the analysis [36,37,38]. The relative distribution of observed and expected −log10 (*p*) values in each trait-associated genomic locus was compared based on a quantile–quantile (Q-Q) plot. The accuracy and robustness of the SNP marker-trait association were determined based on Bonferroni correction (BC) and false-discovery rate (FDR) criteria for multiple testing. In the present study, the *p*-value ≤ 1 × 10^−5^ threshold for significant markers for multiple comparisons was performed at BC and suggested FDR cut-off ≤ 0.05 [39,40,41]. The Q-Q plots of the observed and expected *p*-values were plotted at –log10 (*p*) values to assess the adequacy of a fitted normal straight line to the markers. No significant difference in association and pattern of Q-Q plots were observed with different filter parameters (20% SNP and 20% genotype; 20% SNP and 10% genotype missing data) with different models. The Manhattan plots and Q-Q plots were visualized using GAPIT3 package in R version 4.1.2 (R package GAPIT3, https://CRAN.R-project.org) (accessed on 3 February 2022). Visualization of MTAs on chromosomes by “statsgenGWAS” package in R version 4.1.2.

### 2.7. In Silico SNP Annotation

The candidate genes corresponding to significantly associated MTAs were searched by chickpea genome [42]. The biological functions of annotating genes/transcripts close to the significant SNPs were determined by candidate MTAs with functional proteins related to the grain nutrient and agronomic traits using *UniProt Knowledgebase* database (http://www.uniprot.org/) (accessed on 22 February 2022), QuickGO (http://www.ebi.ac. uk/QuickGO/) (accessed on 22 February 2022).

## 3. Results

### 3.1. Characterization of the Population and the Genetic Relationships

#### 3.1.1. Principal Component Analysis (PCA)

**Figure 1:**
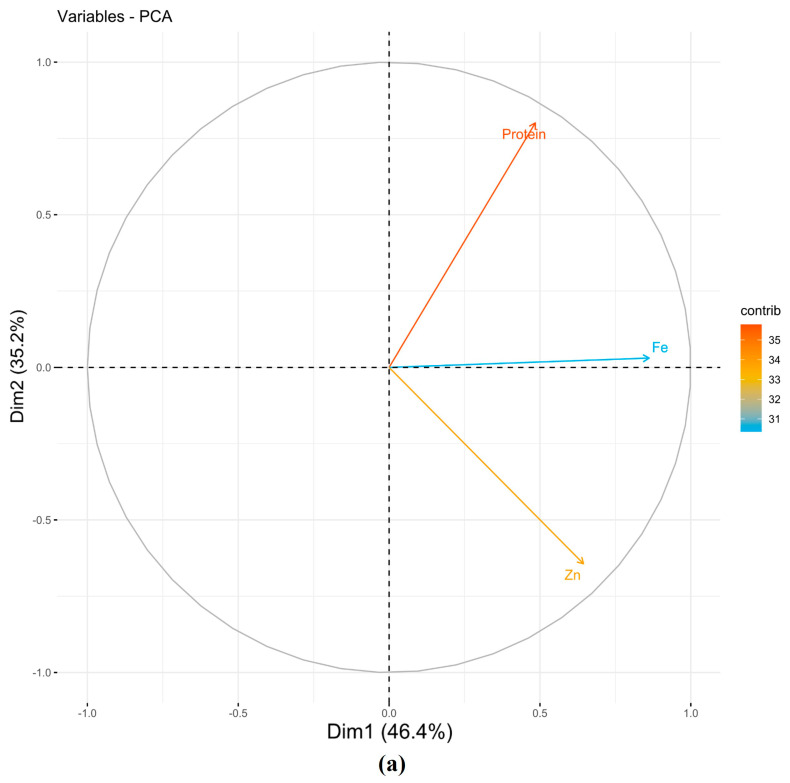

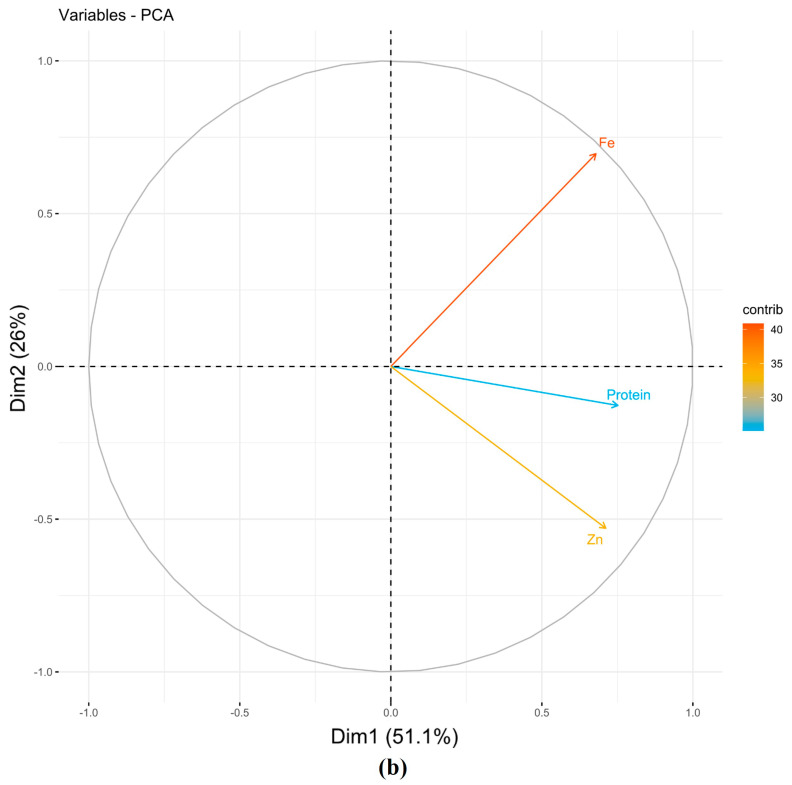

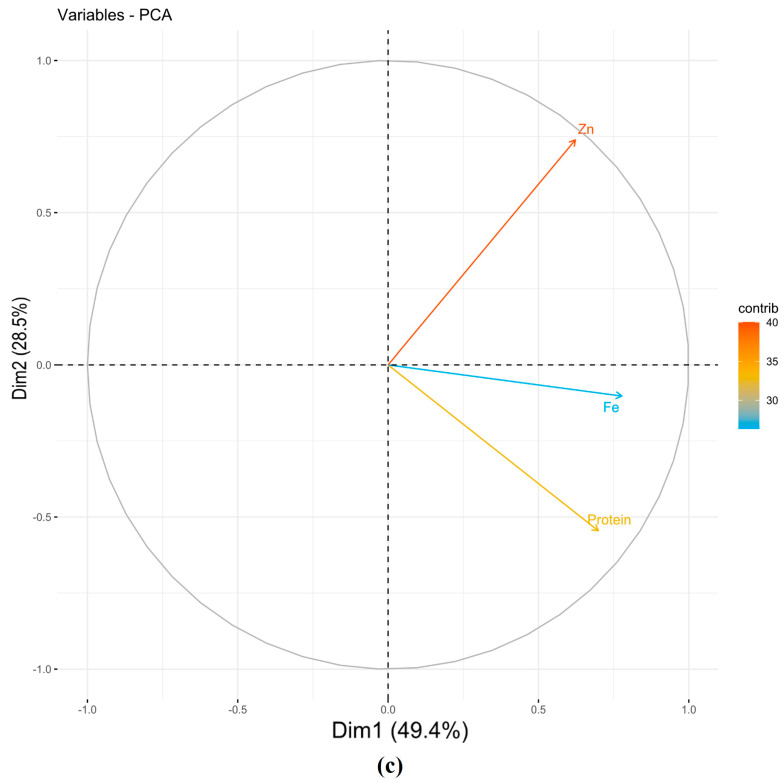
Estimated PCs for grain nutrients (protein, Fe, and Zn) under NSI, NSII, and pooled seasons (**a**–**c**), respectively. Fe, grain iron content; Zn, grain zinc content; NS, normal season.

PCA generated a total of three principal components (PCs), among them PC1 and PC2 (eigenvalues ≥ 1), collectively explained 81.67%, 77.10%, and 77.90% of the total variance under NSI, NSII, and pooled seasons, respectively (Table 1). The PC1 (*x*-axis) explained 46.44%, 51.10%, and 49.40% of the total variance under NSI, NSII, and pooled seasons, respectively. Grain protein, Fe, and Zn correlated positively towards PC1 under NSI, NSII, and pooled seasons, respectively (Figure 1). Similarly, PC2 (*y*-axis) accounted for 35.20%, 26.00%, and 28.5% of the total variation under NSI, NSII, and pooled seasons, respectively. Grain protein and Fe correlated negatively towards PC2 under NSI, NSII (except Fe), and pooled seasons. Similarly, Zn correlated positively except under NSII (Table 1).

#### 3.1.2. Population Structure, Kinship, and Linkage Disequilibrium

**Figure 2:**
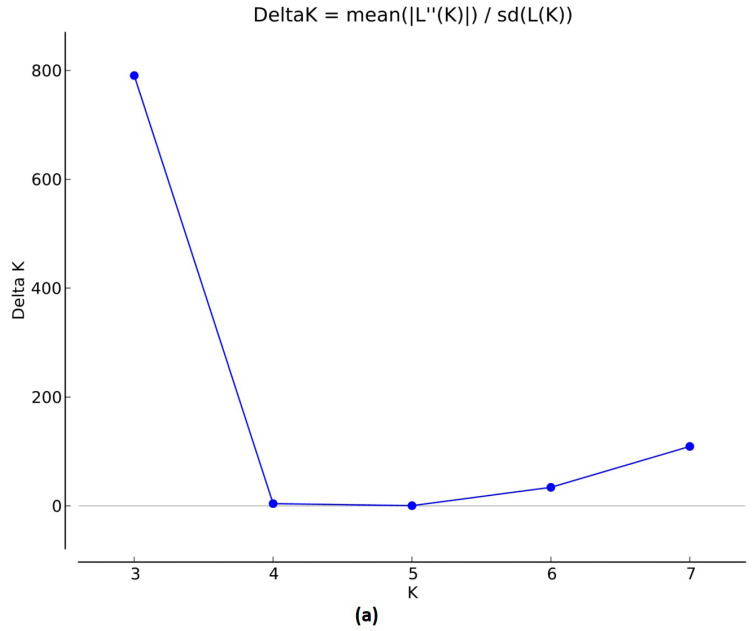

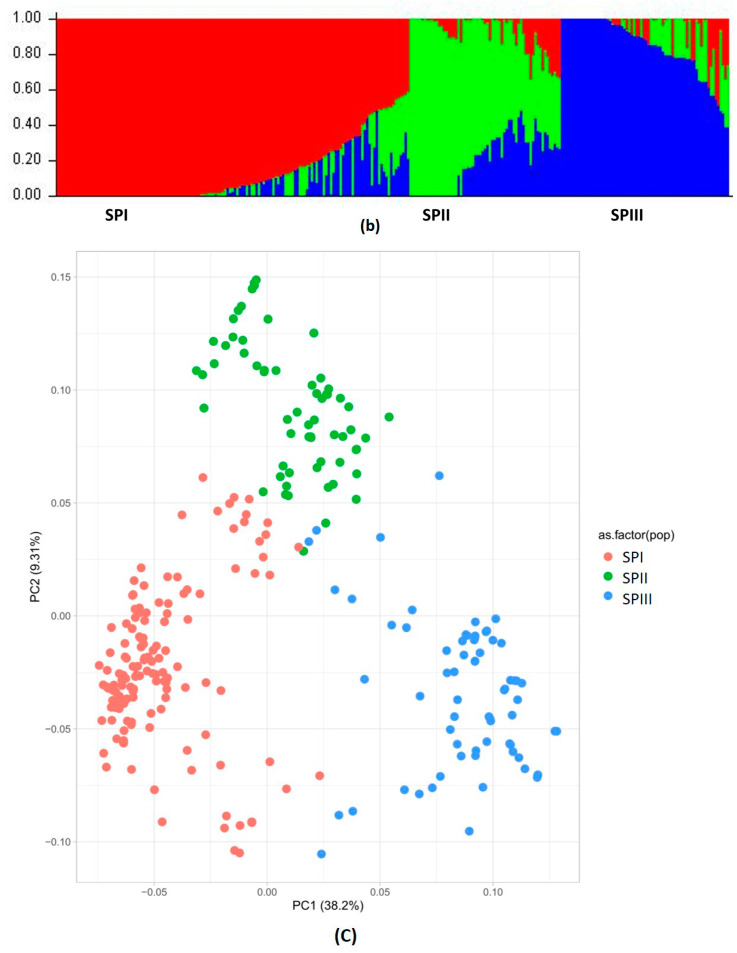

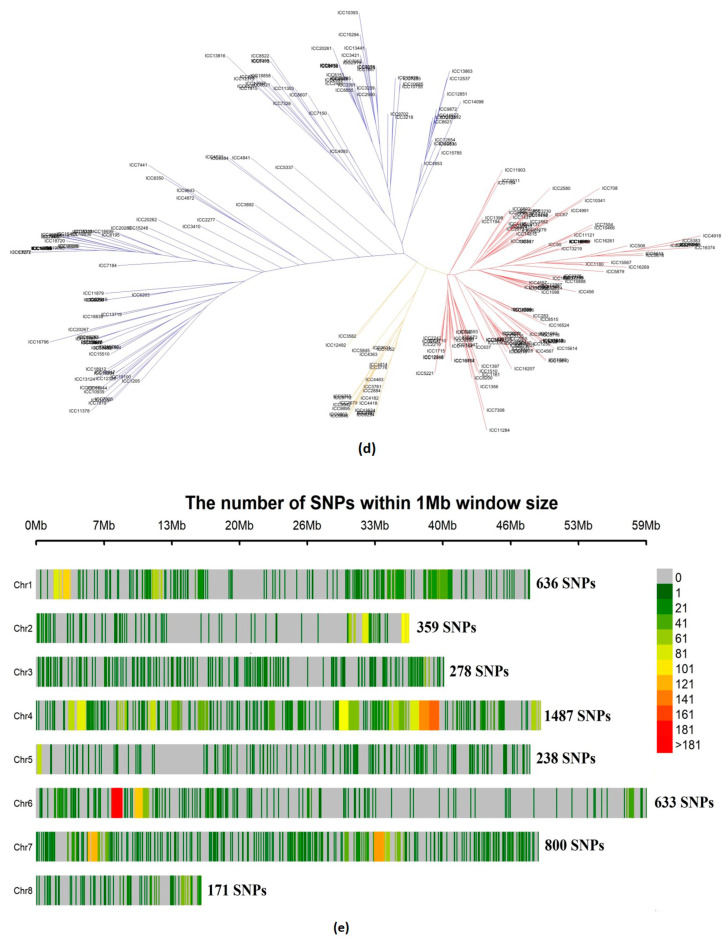
Population structure and phylogenetic analysis. (**a**) Evanno test for the optimum subpopulations (K = 3) using LnP(D)−derived ∆K. (**b**) Population structure inferred into three subpopulations (SP), K = 3, based on ∆K values. (**c**) PCA analysis with 4603 SNP markers. (**d**) Phylogenetic analysis using the neighbor-joining method grouped into three clusters (red, cluster 1; yellow, cluster 2; blue, cluster 3). (**e**) SNP density and distribution across chickpea genome.

The population structure of 280 accessions was analyzed using 4603 high-quality genome-wide SNP arrays. The SNP density and distribution on each chromosome across the chickpea genome are presented in Figure 2e. Model-based simulation of population structure showed the highest peak at K = 3 as the number of sub-populations plotted against (delta) ∆K by Structure Harvester (Figure 2a), thus indicating the presence of three sub-populations (SP1 to SP3) in the reference set (Figure 2b). SP1 was the largest with 147 accessions, including 63 pure types and 84 admixture types that constituted 30% of total accessions (280); SP2 with 63 accessions included 10 pure types and 53 admixtures constituting 18.9% of total accessions; and SP3 with 70 accessions included 21 pure types and 49 admixtures that constituted 17.5% of total accessions. The fixation index (F_st_) was observed as 0.64, 0.60, and 0.62 for SP1, SP2, and SP3 respectively. The allelic frequency of divergence was maximum between SP1 and SP2 at 0.44, and SP3 was 0.29, while it was low (0.19) between SP2 and SP3 (Table 2). PCA revealed three distinct clusters where PC1 explained 38.2% and PC2 9.31% of the total variance (Figure 2c). Similarly, in the unweighted neighbor-joining (NJ) tree method, the accessions were grouped into three clusters. Cluster I had 128 (114 desi and 13 kabuli) accessions, cluster II had 24 (23 desi and 1 kabuli), and cluster III had 128 (61 desi and 67 kabuli) accessions (Figure 2d).

**Figure 3:**
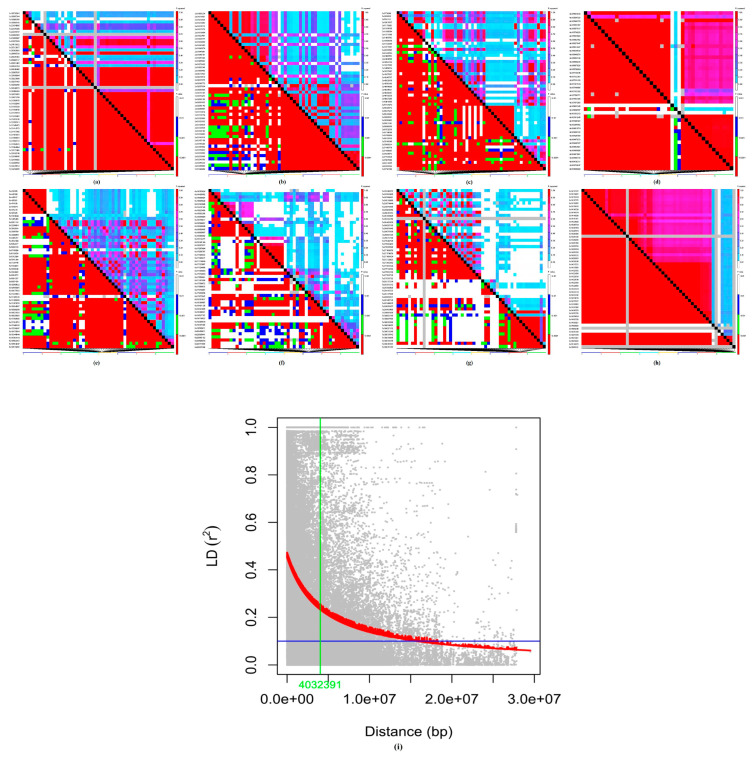
Linkage disequilibrium (LD) patterns on eight chromosomes (**a**–**h**) across the chickpea genome, genotyped with 4603 SNPs (MAF ≥ 0.05). The squared correlation coefficients (*r*^2^) for each pair of markers are presented in the upper triangle and their corresponding tests in the lower triangle. White, *p* < 0.01; blue, 0.01 > *p* > 0.001; green, 0.001 > *p* > 0.0001; and red, *p* < 0.0001. (**i**) LD decay plot with average *r*^2^ value on *y*-axis and the physical distance between markers (bp) on *x*-axis.

The LD pattern across the chickpea genome is presented in Figure 3. LD decay (*r*^2^) based on 4603 SNP array markers in the complete genome was calculated. The *r*^2^_max90_ and the LD_1/2 max90_ percentiles (the physical distance in bp at which LD has decayed) values were obtained at 0.5 and 0.1, respectively. The overall LD decay was relatively low (*r*^2^ > 0.1) at a physical distance of 4032 kb in chickpea germplasm (Figure 3).

#### 3.1.3. Relatedness between Chickpea Accessions

**Figure 4:**
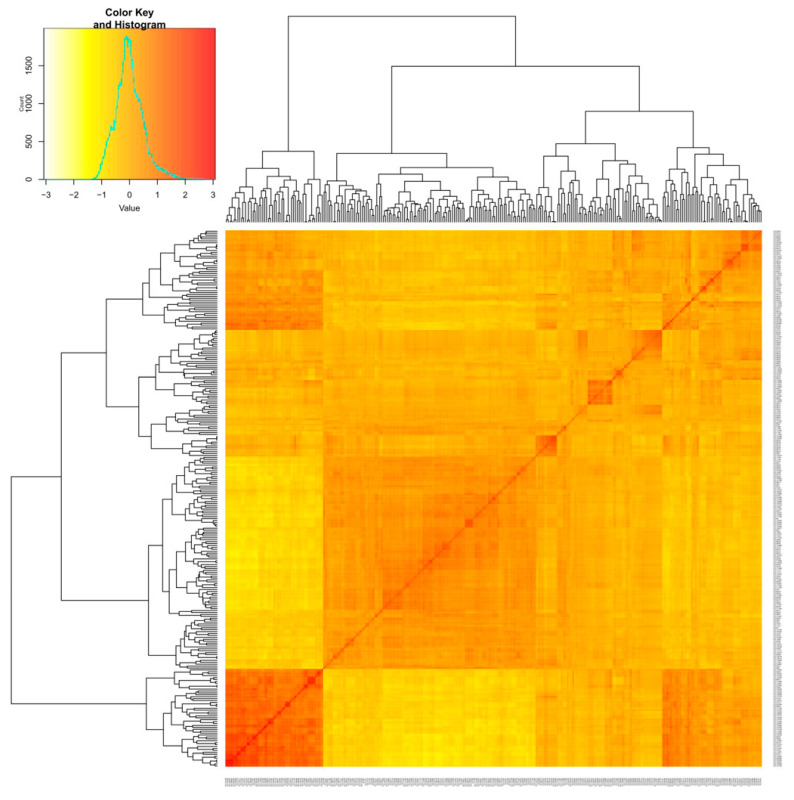
Heat map plot of kinship matrix using average linkage clustering based on SNP markers depicts the existence of three different groups among chickpea accessions.

The kinship coefficient between pairs of chickpea accessions varied from −1.38 to 3 (on a scale of −3 to 3) (Supplementary Table S2). Overall, 14% of the pairs among 280 chickpea accessions had kinship values of ≤ 0.5 (Supplementary Table S1). The genetic distance between accessions varied largely from 0 to 0.83. (Supplementary Table S3). The average genetic distance (GD) between pairs of accessions was 0.40 and 84% of accession pairs with GD of more than 0.25. Kinship matrix obtained from genotyping of SNP markers resulted in three distinct groups (Figure 4) (Supplementary Table S3).

### 3.2. Genome-Wide Association Mapping for Grain Nutrient and Agronomic Traits

A total of 20 (7, 12, and 1 for grain protein, Fe, and Zn) and 46 MTAs (7, 15, and 24 for DFF, DM, 100 SW) were identified from two different GAPIT models, FarmCPU and BLINK, based on significant *p*-values under NSI, NSII, and pooled seasons (Table 3 and Supplementary Tables S4–S6). In NSI, a total of three (one MTA each for grain protein, Fe, and Zn) markers showed significant association (Table 3). The markers S4_4477846, S7_9379786, and S6_7891103 on chr4, 7, and 6 showed highly significant association (*p*-values) with grain protein, Fe, and Zn, respectively (Table 3). Similarly, 14 significant MTAs were observed for DFF (#3), DM (#4), and 100SW (#7) (Supplementary Tables S4–S6). Of which, the markers S1_2001361 (*p* ≤ 6.97 × 10^−7^) on chr1, S4_31996956 (*p* ≤ 1.02 × 10^−9^) on chr4, and S6_26554579 (*p* ≤ 1.63 × 10^−8^) on chr6 showed highly significant association with DFF, DM, and 100 SW traits, respectively.

In NSII, a total of 10 MTAs (4 and 6 for grain protein and Fe) were observed (Table 3). The markers S4_4477846 and S1_2772537 were significantly associated with protein and Fe, respectively. No significant markers were associated with Zn under NSII. Similarly, 18 MTAs were associated with DFF (#4), DM (#5), and 100 SW (#9) (Supplementary Tables S4–S6). S1_15882390 (*p* ≤ 5.54 × 10^−8^), S4_34377824 (*p* ≤ 1.59 × 10^−6^), and S7_32973784 (*p* ≤ 4.79 × 10^−11^) were associated significantly for DFF, DM, and 100 SW, respectively.

In pooled seasons, seven MTAs (five and two for grain protein and Fe, respectively) were significantly associated (Table 3). On chr4 and chr1 with S4_4477846 and S1_11613376 markers, with high *p*-values for grain protein and Fe, respectively. No significant markers were identified for grain Zn content. A total of 14 MTAs were significantly associated with agronomic traits (6 and 8 for DM and 100 SW, respectively) (Supplementary Tables S4–S6). The markers S4_31996956 and S7_32973784 showed highly significant association with DM and 100 SW, respectively (*p* ≤ 1.25 × 10^−6^, *p* ≤ 1.43 × 10^−8^).

**Figure 5:**
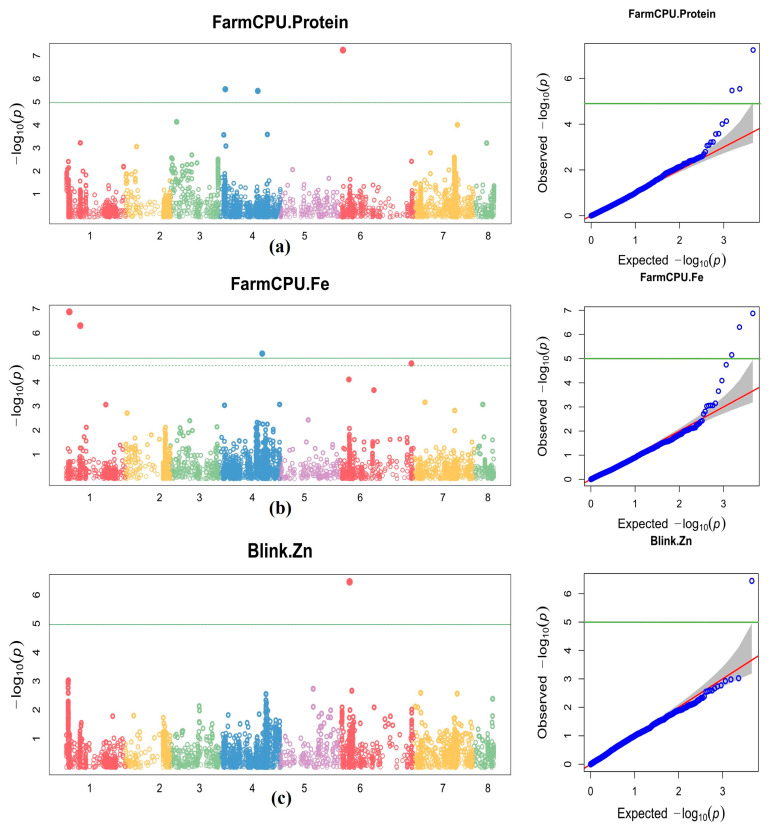
Manhattan plots illustrated significant *p*-value associated with grain protein and Fe in chickpea under NSII by FarmCPU (**a**,**b**) and Zn (NSI) BLINK (**c**) models. Dotted line, suggestive MTAs at FDR cutoff *p* < 0.05; Fe, grain iron content; Zn, grain zinc content; NS, normal season.

**Figure 6:**
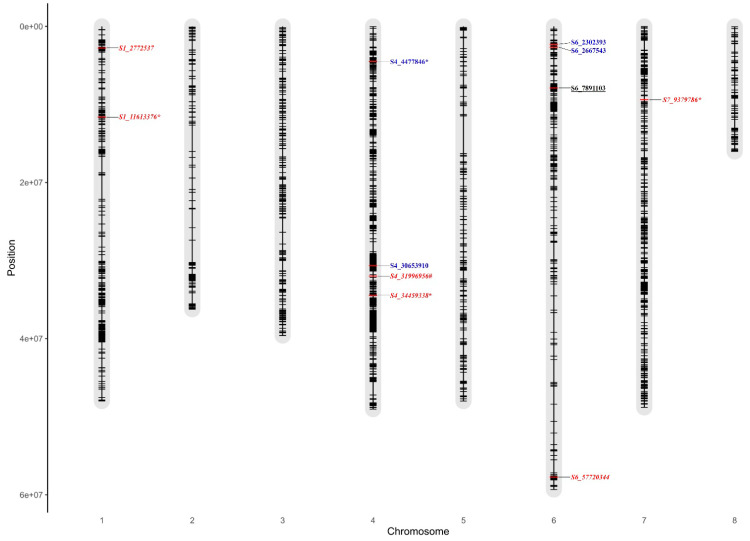
Visualization of significantly associated SNPs (MTAs) along with co-associated SNPs (*) over the seasons on chickpea chromosomes for grain protein (blue, Bold), Fe (red, Bold *Italic*), Zn (black, Bold underline). # Co-associated markers grain Fe and DM. Fe, grain iron content; Zn, grain zinc content.

The marker S4_4477846 on chr4 was co-associated with grain protein under NSI, NSII, and pooled seasons. The markers S1_11613376 and S1_2772537 were co-associated with grain Fe content under NSII and pooled seasons and the S7_9379786 marker under NSI and pooled seasons. The Q-Q plots indicated that observed −log10 (*p*) values of grain protein, Fe, and Zn were in a straight line with a sharply deviated tail close to 1:1 line with the expected values under NSI, NSII, and pooled seasons (Figure 5 and Supplementary Figures S1 and S2). The S6_57417458 marker was co-associated with DFF under NSI and NSII. S6_5707534 and S4_31996956 markers were found to be correlated for DM under NSII and pooled seasons. S7_32973784, S6_26554579, S6_6142886, and S2_191229 markers were found to be co-associated with 100 SW under NSI, NSII, and pooled seasons (Table 3). Visualization of MTAs on chromosomes for nutrient and agronomic traits is presented in Figure 6 and Supplementary Figure S6. Co-association of markers between the nutrient and agronomic traits were observed among the FarmCPU, BLINK, and SUPER models (Table 3, Supplementary Figures S3–S5). The marker S4_31996956 was co-associated with grain Fe and DM (Table 3). Similarly, S1_15882390 (*p* ≤ 6.97 × 10^−6^) and S4_34377824 (*p* ≤ 6.13 × 10^−6^) markers were co-associated with DFF and DM. Manhattan plots with significant SNPs for grain nutrient and agronomic traits are presented in Figure 5 and Supplementary Figures S1–S6.

### 3.3. Annotation of Associated SNPs

The structural and functional impact of associated SNPs were identified by comparing SNPs’ relative position to the annotated chickpea genome. The significant MTAs for nutrient and agronomic traits were retrieved, annotated based on gene ontology (GO), and categorized into cellular component, biological process, and molecular function. Nineteen SNPs were functionally annotated, while the remaining markers had putative gene function (Supplementary Table S7). Among the total annotated SNPs, 45.7% were intergenic, 17.4% were intronic, 10.9% synonymous variants, 13% were non-synonymous (missense and non-sense variants), and 13% were putative. The annotated SNPs with different gene functions were related to transferase, synthases, transporters, protein kinases, zinc finger protein, ion gated channels, repressor proteins, DNA-binding proteins, peptidase, and many more functions involved in plant metabolism along with Fe and protein homeostasis. Genes associated with nutrient and agronomic traits are presented in Supplementary Table S7.

## 4. Discussion

Malnutrition in various forms and rising hunger are major obstacles for achieving food and nutritional security. Uncovering genomic regions/genes associated with nutritional traits in chickpea will hasten the development of biofortified cultivars to address malnutrition. In chickpea, understanding the genetic diversity of essential grain nutrients such as protein, Fe, and Zn as well as their association to yield-related traits is critical for expediting biofortified variety development. The recent studies on chickpea germplasm revealed a wide phenotypic variation for grain protein and Fe and Zn content [15,16]. These studies help to identify diverse sources of donors for improving nutrient levels in the newly developed varieties.

Large-scale informative trait-specific SNP markers/arrays were developed and deployed in chickpea to assist breeding programs on a genome-wide scale [22,43,44,45]. The “Axiom^®^*Cicer*SNP Array”, a high-throughput single-nucleotide polymorphism (SNP) genotyping technology, has aided the construction of dense genetic maps to advance genetics and breeding efforts [45]. The SNP genotyping arrays are efficient, user-friendly, and cost-effective, providing robust and reliable data with fewer missing values in comparison to other genotyping platforms. SNPs arrays with medium-to-high density were also used in various plant species as well as in chickpea [31,45].

The number of markers required for GWAS analysis is determined by the extent of LD across the genome. The LD value of a population will suggest the evolutionary changes that aid in the more precise mapping of quantitative traits such as grain protein, Fe, and Zn and also provide insights into the joint evolution of the linked sets of genes. The lower the LD across the genome, the higher the density of markers required for better mapping resolution and vice versa [46]. The significant marker-trait association depends on the pattern and extent of LD across the genome [47]. In the present study, LD (r^2^) decay was slow across the genome. LD decay occurred at a physical distance of 4032 kb, indicating a higher LD extent in chickpea reference set across the genome (Figure 3). Chickpea landraces exhibit extended LD, indicating a very slow rate of decay [48]. A higher LD was observed in chickpea due to low effective recombination rates [15,49]. In comparison to cross-pollinated crops, LD decay was observed to be slower in self-pollinated crops [15,50]. The extent of LD decay in the association panel of chickpea was observed at 200–300 kb [51] and at 5 cM in chickpea reference set [20] and for cowpea at 1.4 Mb [52]. The extent of LD can vary due to the complexity and size of the genome and marker number [53].

Population structure analysis revealed three subgroups/subpopulations with certain admixtures, indicating that common gene/allelic combinations continue among the chickpea accessions. The expected heterozygosity among the subpopulation was relatively low (Table 2). This low level of heterozygosity based on SNPs suggests that accessions in the present study were homozygous and close to being inbred. The genetic diversity based on net nucleotide distance between SPs was relatively high (Table 2). Similarly, low expected heterozygosity and high genetic divergence among the subpopulations were reported in chickpea (breeding lines and landraces) [54]. The observed three SPs were SP1 (130 desi and 17 Kabuli accessions), SP2 (45 desi and 18 Kabuli), and SP3 (24 desi and 46 Kabuli) accessions. The results of SPs were similar to the clusters grouping pattern by the neighbor-joining (NJ) method. These observations could be owing to a stronger effect of geographical origin and adaptive environment on assigning accessions to a specific population group rather than cultivars. The diversifying and non-recurrent germplasm (desi, kabuli) lines were exploited in the breeding program, and complicated domestication and breeding history along with high-adaptive selection patterns could lead to admixtures across accessions reported [20,28,44,55].

The extent of relatedness between chickpea accessions was determined by using kinship values derived between pairs of accessions. Kinship values near zero imply unrelated accessions, whereas those near 0.5 or higher (14% of the accession pairs) indicate full sibs or highly similar germplasm. A highly variable genetic distance between the accessions was observed (0 to 0.83). This result is notable, as previous studies report the narrow genetic base in chickpea germplasm [56]. The average genetic distance (GD) between pairs of accessions was 0.40, with 84% of accession pairs with GD of more than 0.25, indicating the presence of large genetic variability among chickpea accessions for the target traits. Farahani et al. [54] reported a large genetic variability (88%) with GD > 0.25 in landraces and breeding lines of chickpea.

The cluster pattern by neighbor-joining tree grouped the chickpea accessions into three cluster groups, which concurs with the population structure and PCA analysis reports. These findings imply that hybridization between inter-cluster accessions could result in nutrient-rich chickpea cultivars. However, certain clusters had a mix of kabuli, desi, landraces, and advanced cultivars, and some accessions were grouped in distinct clusters. This could be due to the employment of the same accessions in paternal crossing as well as the domestication and selection of similar chickpea accessions over centuries, all of which had a significant impact on global chickpea genetic structure, resulting in genotypic admixture, as shown in the current study and earlier studies in chickpea [15,54,55,57,58].

A total of 66 MTAs were identified for nutrient and agronomic traits under NSI, NSII, and pooled seasons using FarmCPU and BLINK models. To overcome the limitations of single-locus GWAS, these multi-locus association models were used over other models to identify MTAs with maximum statistical power and high prediction to avoid false-positive and false-negative values. Solid lines with lambda inflation factor (λ = 0.87–1.33) in quantile–quantile (Q-Q) plots confirmed the suitability of the multi-locus association models (Figure 5, Supplementary Figures S1–S5) [59]. Furthermore, the Bonferroni correction (BC) and false-discovery rate (FDR) criteria were used to reduce false positives caused by multiple testing. For grain protein, MTAs were significantly associated on chr4 and 6. One marker, S4_4477846 (average *p* ≤ 1.38 × 10^−6^), was co-associated under NSI, NSII, and pooled seasons for grain protein. MTAs for grain Fe content were significantly associated on chr1, 4, 6, and 7 over the seasons. No correlated markers were observed between NSI and NSII for grain Fe content; however, one marker was found common between NSI and pooled seasons and three markers under NSII and pooled seasons (Table 3). These stable MTAs for grain protein and Fe are valuable resources for improving nutrient quality in chickpea cultivars. However, one MTA (S6_7891103, *p* ≤ 3.52 × 10^−7^) was observed for grain Zn content under NSI but no MTAs under NSII and pooled seasons, which indicates the significant influence of seasons on the Zn content in the current population. Earlier studies also reported location/season-specific MTAs and no common MTAs between locations/seasons for grain Fe and Zn content in chickpea [11,15], pea [26].

Four significant MTAs for grain protein and Fe were identified on chr4. Based on the position of markers, a physical map was generated to compare the significant markers between the current study and Samineni et al. [15]. The latter study reported 10 MTAs for grain protein on chr4 (non-stress) and 6 on chr6 (drought) conditions. Two markers, namely S4_2624940 and S4_5775736, were found highly significant among the other markers for grain protein content on chr4 [15], and interestingly, one highly significant SNP for grain protein content in the current study was positioned between these two SNPs. However, loci on chromosomes 1, 3, and 6 were also found to contribute for protein content [60]. Thus, the current investigation identified additional markers within the genomic regions on chr4 for grain protein and Fe content, which will help in marker development and further use in the selection process. Current and previous studies identified markers for Fe content on chr1, 4, 6, and 7 of chickpea [11,15,28,61]. This also suggests the presence of a high-LD pattern and extent across the chickpea genome (Figure 3). This indicates the complex and quantitative nature of these traits. One significant MTA for grain Zn was found on chr6. On the contrary, earlier studies in chickpea reported MTAs on chr1, 4, and 7 [11]; chr3, 4, 5, and 7 [28]; chr2, 4, and 5 [61]; and chr1, 4, and 7 [15]. This indicates chr4 was harboring the genomic regions as well as significant co-localized MTAs for grain protein and Fe across diverse genetic backgrounds in chickpea [11,15,28,30,61]. The significant tightly linked MTAs on chr4 could be used for further validation in diverse populations and identification of candidate genes for early generation selections in the breeding pipeline.

Significant markers for DFF were found on chr1, 4, and 6 and well-corresponded with previous studies in chickpea [18,62]. For DM, significant associations were observed on all the chromosomes except on chr3, 5, and 7 and were in accordance to earlier studies in chickpea [45]. Similarly, significant markers for 100 SW were found on all the chromosomes except chr2 and 4. However, earlier studies found the associated markers and QTLs for 100 SW on all the chromosomes in chickpea [22,62,63,64]. This indicates the complex genetic architecture of these traits in chickpea. The identified co-located markers over seasons in the present study were novel and found no similarity with earlier reports in chickpea. These markers will be useful for the simultaneous improvement of traits using marker-assisted selection.

Identification of candidate genes is essential to understand the molecular mechanism underlying the particular trait. In the current study, functionally annotated SNPs were involved in binding, transporters, transcription factors, transmembrane proteins, transferases, zinc finger protein, protein glycosylation, phosphorylation, and many more functions (Supplementary Table S7). The markers S6_2327550 and S6_57720344 corresponding to pentatricopeptide repeat-containing proteins (PPR) and VRN1 genes were shown to have a specific role in seed development, flowering, and Fe homeostasis in different crops [64,65]. S6_45756828 corresponded to CCCH-type zinc finger proteins in the regulation of plant growth, developmental processes, and environmental responses in chickpea [66]. Similarly, two markers (S6_57854709, S6_6142886) corresponding to receptor-like kinases and auxin signaling factors play crucial roles in cell division and cell expansion in the meristematic tissue [67]. Zinc finger protein has a function in Fe and Zn homeostasis [61] (Supplementary Table S7), thus indicating the role of these associated markers in plant growth and grain nutrient homeostasis in chickpea. These markers require further validation, characterization, and gene cloning to elucidate the exact role of these genes in chickpea. The significantly associated SNPs for grain nutrients can be used as informative molecular tags in marker-assisted breeding to enhance grain protein and Fe and Zn content in chickpea cultivars.

## 5. Conclusions

In this study, the genetic basis of grain protein, Fe and Zn content in chickpea reference set was dissected with a mid-density SNP array through GWAS analysis. The population structure, kinship, and neighbor-joining method grouped accessions into three subpopulations. LD was extensive across the genome, and LD decay was observed at a physical distance of 4032 kb. GWAS models (FarmCPU and BLINK) with high statistical power have enormous potential to accelerate breeding strategies to improve the nutritional quality of chickpea, as they allow breeders to make the selection based on the most significant marker-trait associations. Twenty and forty-six SNP markers were found to be associated significantly with the grain nutrient and agronomic traits in the chickpea reference set over the seasons along with co-localized markers between the seasons. The associated markers annotation resulted in genes regulating the functions of ion binding, transporters, transmembrane proteins, transferases, zinc finger protein, protein glycosylation, phosphorylation, and many more functions. The associated markers can be used as informative molecular tags in marker-assisted selection to enhance grain nutritional quality in chickpea.

## Figures and Tables

**Table 1:** PCA estimation, eigenvalue, and their percent variance contribution for grain nutrients.

PCs	Protein	Fe	Zn	Eigenvalues	Variance %	Cumulative Variance
NSI						
PC1	0.41	0.73	0.55	1.39	46.44	46.44
PC2	−0.78	−0.03	0.63	1.06	35.23	81.67
PC3	0.47	−0.68	0.56	0.55	18.33	100.00
NSII						
PC1	0.61	0.55	0.57	1.53	51.09	51.09
PC2	−0.14	0.79	−0.60	0.78	26.05	77.13
PC3	−0.78	0.28	0.56	0.69	22.87	100.00
Pooled seasons						
PC1	0.57	0.64	0.51	1.48	49.37	49.37
PC2	−0.59	−0.11	0.80	0.85	28.48	77.85
PC3	0.57	−0.76	0.31	0.66	22.15	100.00

Fe, grain iron content; Zn, grain zinc content; NS, normal season.

**Table 2:** Genetic divergence among (net nucleotide distance) and within accessions from population structure.

Population	Net Nucleotide Distance		Expected Heterozygosity	% of Membership	Mean Fixation Index (F_st_)
	SPII	SPIII			
SPI	0.44	0.29	0.23	0.29	0.63
SPII		0.19	0.19	0.49	0.64
SPIII			0.17	0.22	0.61

SP, subpopulation.

**Table 3:** MTAs for nutrient traits under NSI, NSII, and pooled seasons by BLINK and FarmCPU models.

SNP	Model	Chromosome	Position	Allele 1	Allele 2	MAF	*p*-Value
Protein, NSI							
S4_4477846 *	BLINK	4	447,7846	A	G	0.49	2.52 × 10^−7^
NSII							
S4_4477846	BLINK	4	447,7846	A	G	0.49	2.42 × 10^−9^
S6_2302393	FarmCPU	6	2,302,393	T	C	0.39	5.83 × 10^−8^
S4_4477846	FarmCPU	4	447,7846	A	G	0.49	2.76 × 10^−6^
S4_30653910	FarmCPU	4	30,653,910	A	G	0.31	3.35 × 10^−6^
Pooled							
S4_4477846	BLINK	4	4,477,846	A	G	0.49	4.90 × 10^−9^
S6_2667543	BLINK	6	2,667,543	C	A	0.47	2.43 × 10^−6^
Fe, NSI							
S7_9379786 #	BLINK	7	9,379,786	T	C	0.44	7.42 × 10^−9^
NSII							
S1_2772537	FarmCPU	1	2,772,537	C	A	0.31	2.89 × 10^−7^
S4_34459338 *	BLINK	4	34,459,338	C	G	0.34	5.09 × 10^−7^
S1_11613376 *	FarmCPU	1	11,613,376	T	A	0.42	6.96 × 10^−7^
S1_11613376	BLINK	1	11,613,376	T	A	0.42	2.15 × 10^−6^
S6_57720344	FarmCPU	6	57,720,344	C	T	0.08	5.56 × 10^−6^
S4_34459338	FarmCPU	4	34,459,338	C	G	0.34	1.12 × 10^−5^
Pooled							
S7_9379786	BLINK	7	9,379,786	T	C	0.44	4.13 × 10^−6^
S4_31996956	BLINK	4	31,996,956	A	C	0.14	4.52 × 10^−6^
S1_11613376	FarmCPU	1	11,613,376	T	A	0.42	1.01 × 10^−6^
S1_2772537	FarmCPU	1	2,772,537	C	A	0.31	2.34 × 10^−6^
S7_9379786	FarmCPU	7	9,379,786	T	C	0.44	3.03 × 10^−6^
Zn, NSI							
S6_7891103	BLINK	6	7,891,103	G	A	0.29	3.52 × 10^−7^

Fe, grain iron content; Zn, grain zinc content; NS, normal season; MAF, minor allele frequency. * Marker significantly associated among BLINK, FarmCPU, and SUPER models. # Marker significantly associated among BLINK and FarmCPU.

## Data Availability

Data available within the article and Supplementary Materials.

## Supplementary Materials

The following supporting information can be downloaded at: https://www.mdpi.com/article/10.3390/cells11152457/s1, Figure S1: Manhattan plots illustrated significant p-value associated with grain protein content in chickpea under NSI; Figure S2: Manhattan plots illustrated significant p-value associated with grain Fe content in chickpea under NSII; Figure S3: Manhattan plots illustrated significant p-value associated with DFF (a), DM (b), 100 SW (c,d) in chickpea under NSI; Figure S4: Manhattan plots illustrated significant p-value associated with DM (a,c), 100 SW (b,d) in chickpea under pooled seasons; Figure S5: Manhattan plots illustrated significant p-value associated with DFF (a,d), DM (b), 100 SW (c) in chickpea under NSII. Figure S6: Visualization of significantly associated SNPs (MTAs) along with co-associated SNPs (*) over the seasons on chickpea chromosomes for DFF (Pink, Bold Italic), DM (Yellow, Bold underline), 100 SW (Maroon, Bold); Table S1: Chickpea reference set accessions with origin and biological status; Table S2: Kinship matrix among the accessions by GAPIT software; Table S3: Genetic distance among the accessions by TASSEL software; Table S4: MTAs for DFF by FarmCPU and BLINK models under NSI, NSII, and pooled seasons; Table S5: MTAs for DM by FarmCPU and BLINK models under NSI, NSII, and pooled seasons; Table S6: MTAs for 100 SW by FarmCPU and BLINK models under NSI, NSII, and pooled seasons; Table S7: Annotation of associated SNPs for nutrient and agronomic traits.
