# Supplementary material for: Genome-Wide Association Analysis Reveals Trait-Linked Markers for Grain Nutrient and Agronomic Traits in Diverse Set of Chickpea Germplasm
Source: Cells. 2022 Aug 8;11(15):2457. doi: 10.3390/cells11152457 (PMC9367858; doi:10.3390/cells11152457)
Supplement: Supplementary file 1 [file cells-11-02457-s001.zip › Supplementary Figs GWAS.pdf]

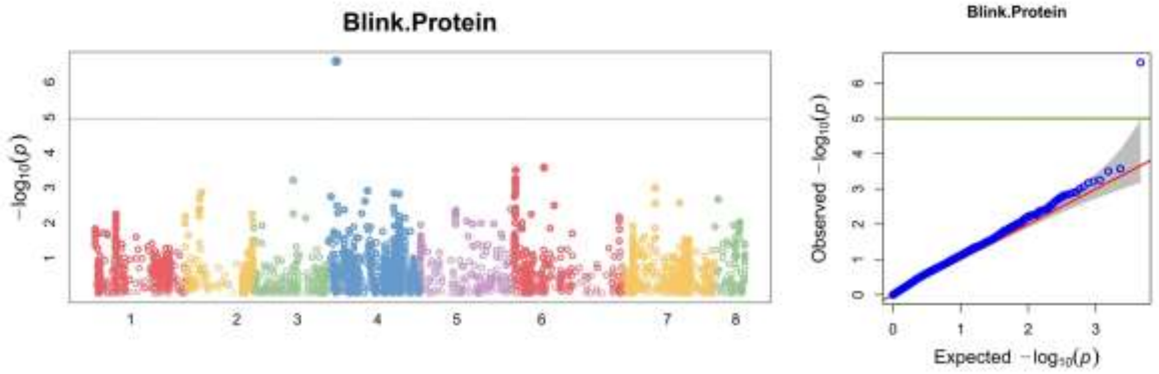

Supplementary Figure S1. Manhattan plots illustrated significant P-value associated with grain protein content in chickpea under NSI.

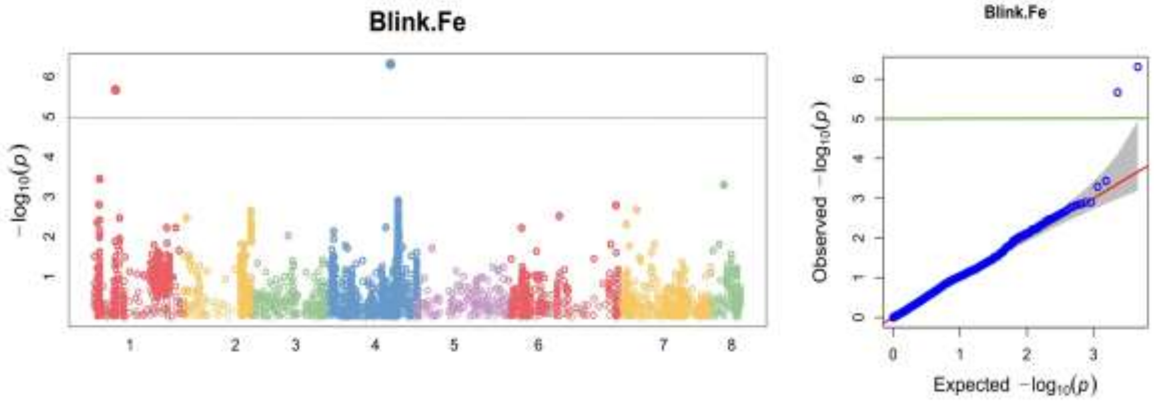

Supplementary Figure S2. Manhattan plots illustrated significant P-value associated with grain Fe content in chickpea under NSII.

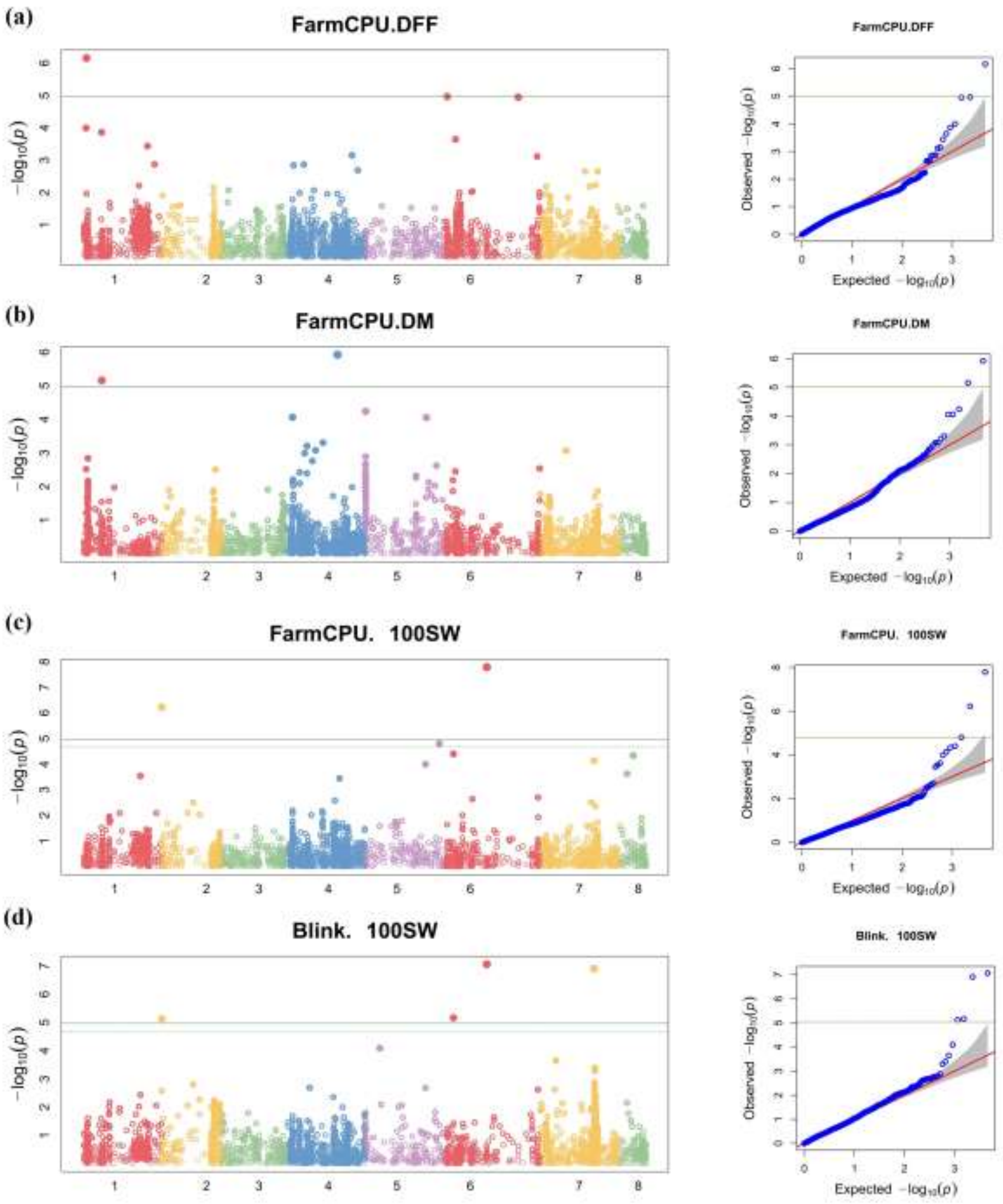

Supplementary Figure S3. Manhattan plots illustrated significant P-value associated with, DFF (a), DM (b), 100 SW (c and d) in chickpea under NSI. Dotted line-suggestive MTAs at FDR cutoff P<0.05.

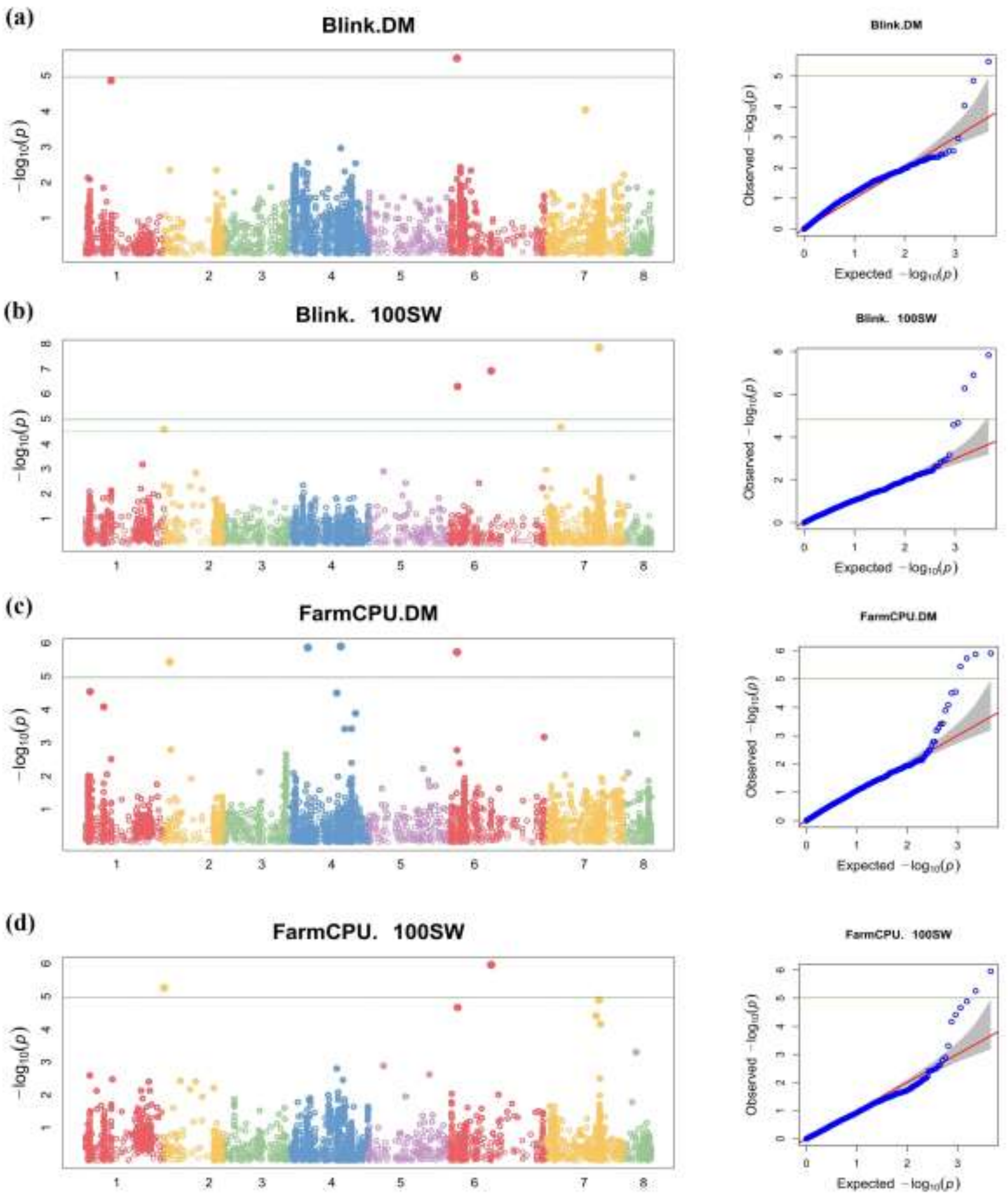

Supplementary Figure S4. Manhattan plots illustrated significant P-value associated with DM (a and c), 100 SW (b and d) in chickpea under pooled seasons. Dotted line-suggestive MTAs at FDR cutoff P<0.05.

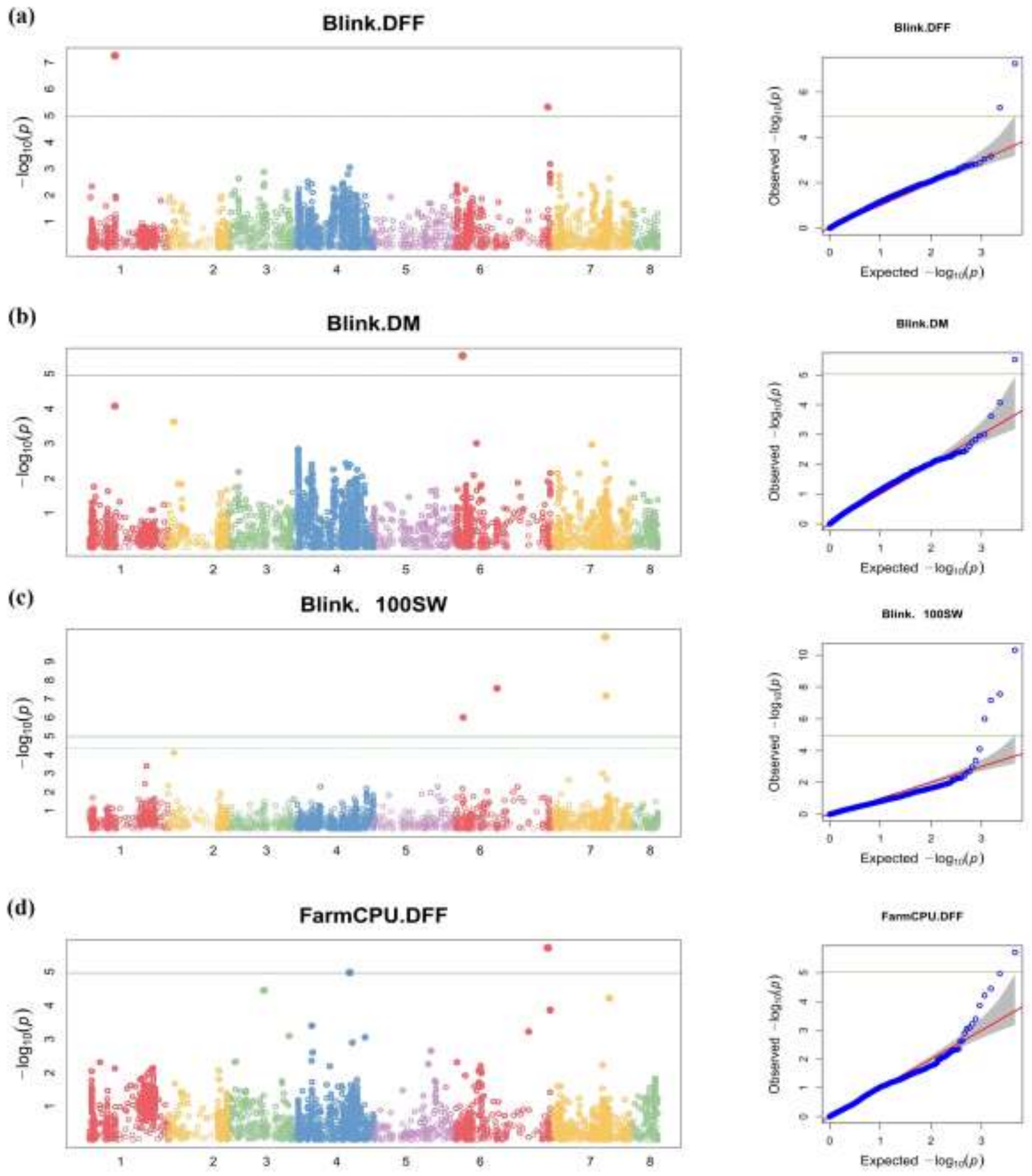

Supplementary Figure S5. Manhattan plots illustrated significant P-value associated with DFF (a and d), DM (b), 100 SW (c) in chickpea under NSII. Dotted line-suggestive MTAs at FDR cutoff $P<0.05$.

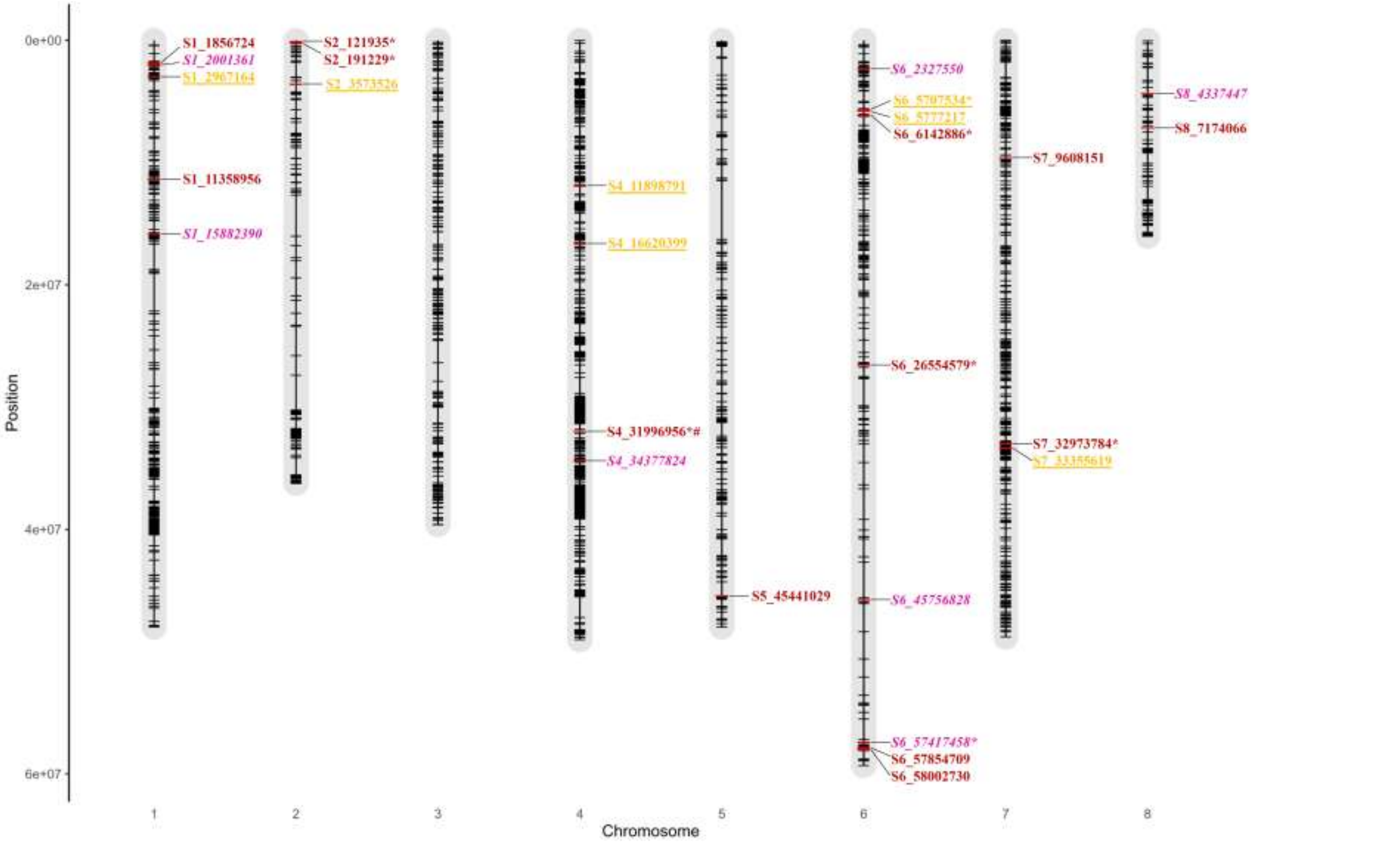

Supplementary Figure S6. Visualization of significantly associated SNPs (MTAs) along with co-associated SNPs (*) over the seasons on chickpea chromosomes for DFF (Pink- **Bold** *Italic*), DM (Yellow-**Bold** underline), 100 SW (Maroon- **Bold**).
